# Thermosensitive Liposome-Mediated Drug Delivery in Chemotherapy: Mathematical Modelling for Spatio–temporal Drug Distribution and Model-Based Optimisation

**DOI:** 10.3390/pharmaceutics11120637

**Published:** 2019-11-29

**Authors:** Yu Huang, Boram Gu, Cong Liu, Justin Stebbing, Wladyslaw Gedroyc, Maya Thanou, Xiao Yun Xu

**Affiliations:** 1 Department of Chemical Engineering, Imperial College London, London SW7 2AZ, UK; yu.huang11@imperial.ac.uk (Y.H.); b.gu13@imperial.ac.uk (B.G.); cliu1106@gmail.com (C.L.); 2 Department of Surgery and Cancer, Imperial College London, London W12 0NN, UK; j.stebbing@imperial.ac.uk; 3 Department of Experimental Medicine, Imperial College London, London W2 1NY, UK; w.gedroyc@imperial.ac.uk; 4 School of Cancer and Pharmaceutical Sciences, King’s College London, London SE1 9NH, UK; maya.thanou@kcl.ac.uk

**Keywords:** thermosensitive liposome, drug delivery, multi-compartmental model, mathematical model, optimisation

## Abstract

Thermosensitive liposome-mediated drug delivery has shown promising results in terms of improved therapeutic efficacy and reduced side effects compared to conventional chemotherapeutics. In order to facilitate our understanding of the transport mechanisms and their complex interplays in the drug delivery process, computational models have been developed to simulate the multiple steps involved in liposomal drug delivery to solid tumours. In this study we employ a multicompartmental model for drug-loaded thermosensitive liposomes, with an aim to identify the key transport parameters in determining therapeutic dosing and outcomes. The computational model allows us to not only examine the temporal and spatial variations of drug concentrations in the different compartments by utilising the tumour cord concept, but also assess the therapeutic efficacy and toxicity. In addition, the influences of key factors on systemic plasma concentration and intracellular concentration of the active drug are investigated; these include different chemotherapy drugs, release rate constants and heating duration. Our results show complex relationships between these factors and the predicted therapeutic outcome, making it difficult to identify the “best” parameter set. To overcome this challenge, a model-based optimisation method is proposed in an attempt to find a set of release rate constants and heating duration that can maximise intracellular drug concentration while minimising systemic drug concentration. Optimisation results reveal that under the operating conditions and ranges examined, the best outcome would be achieved with a low drug release rate at physiological temperature, combined with a moderate to high release rate at mild hyperthermia and 1 h heating after injection.

## 1. Introduction

The efficacy of conventional chemotherapy is limited by poor distribution of therapeutic agents in tumour tissues and dose-limiting side effects. Liposomes loaded with anticancer agents have been developed as drug carriers to reduce systemic cytotoxicity, and yet their efficacy is limited by insufficient release of contents at the tumour site [1]. Thermo-sensitive liposomes (TSLs) in combination with mild hyperthermia (HT; ~42 °C) have emerged as an attractive option to improve tumour drug delivery owing to the ability of the TSLs to release their payload at the targeted tumour site upon localised heating to mild HT (40–44 °C) [2,3]. Experimental studies have shown that focused ultrasound is not only effective in triggering drug release from TSLs, but also beneficial through increasing the hydrostatic permeability of blood vessels and consequently enhancing the diffusion rate of drug, leading to accelerated transport and better penetration of drug in the targeted tumour. The underlying mechanism of enhanced vascular permeability in tumours upon the application of focused ultrasound is still unknown, but it has been suggested that hyperthermia can cause an increase in macromolecular transport [4]. The use of TSLs in conjunction with focused ultrasound-hyperthermia in chemotherapy can potentially reduce the required dose, the risk of side effects and enhance therapeutic effectiveness through local drug release and improving the tumour microenvironment.

Over the last decade, computational modelling has served as an effective tool to assess the therapeutic potential of chemotherapy using TSLs and to provide information on intratumoural distribution of anticancer agents [5,6,7,8,9,10,11]. Compared to conventional delivery of free anticancer agents and stealth liposomes, evaluating the efficacy of TSLs involves additional complexities due to heat transfer phenomena in tumour tissues induced by focused ultrasound, which gives rise to spatio–temporal variations of temperature and temperature-dependent blood perfusion and vascular permeability. In this context, mathematical models can be particularly useful in elucidating the complex interactions between HT exposure and transport steps, and in complementing experimental research through parametric sensitivity analysis and optimisation.

Several computational studies have been carried out to investigate temporal profiles of anticancer drug concentrations in response to a uniform temperature rise using a drug transport model without heat transfer [7,8]. Gasselhuber et al. [6,9] improved the mathematical model by incorporating Penne’s heat transfer equation, temperature-dependent perfusion rates and release kinetics in order to predict the spatio–temporal distribution of temperature as well as heterogeneous profiles of drug concentration. More recently, Zhan et al. [10] reported a multiphysics modelling framework for TSL-mediated drug delivery by coupling high intensity focused ultrasound acoustics with spatially revolved drug transport models. Their model also incorporated temperature-dependent tumour and drug properties. However, the aforementioned studies have mainly focused on macroscopic distributions of TSLs and released agents at the tumour site while neglecting micro-scale variations in tumour interstitial space between blood vessels. Drug distributions at the micro-scale may hold important information on transport rates through membranes which hinder drug penetration. Due to the abnormal and heterogeneous tumour vasculature, incorporating realistic tumour vasculature geometry in a spatially-resolved drug transport model poses a significant challenge. Therefore, a mechanistic modelling approach based on the tumour cord concept has been widely adopted to describe micro-scale transport phenomena of a variety of blood-borne molecules, including oxygen, free drug, macromolecules and nanoparticles [12,13,14,15].

Our current study aims to investigate drug transport from microvessels to the surrounding tumour tissue for a TSL drug delivery system and to assess the treatment efficacy with different TSL properties and HT duration by means of computational simulation. An integrated computational model has been developed, which consists of a multi-compartmental model to describe the pharmacokinetics (PK) of TSLs and encapsulated agents and a spatially-resolved transport model to predict the spatio–temporal distributions of drug concentration in tumour interstitial space. Our computational results show qualitative agreement with experimental data obtained on a murine cancer model using near infrared fluorescence labelled thermosensitive liposomes [16]. Detailed comparisons are made between predictions for two anticancer agents: Doxorubicin (DOX) and topotecan (TOP). The influences of drug release rate of TSL and heating duration are examined. Finally, model-based optimisation is performed to maximise the intracellular drug concentration while minimising the systemic drug concentration, which are indicative of therapeutic effectiveness and cytotoxicity, respectively. Three parameters are chosen to be optimised: Drug release rate at normal physiological temperature, drug release rate at mild hyperthermia and heating duration. The optimisation framework provides a potentially useful tool for selecting liposome properties and therapeutic protocols in order to design safer and more effective treatments.

## 2. Methods

**Figure 1:**
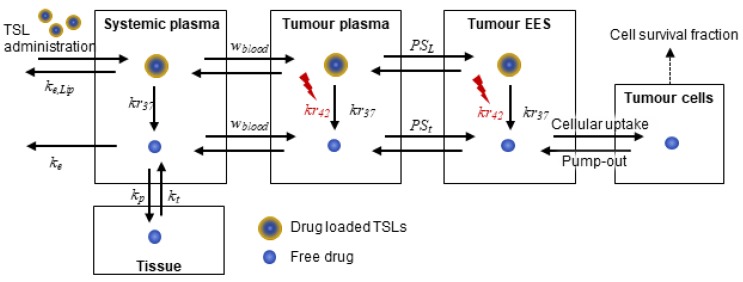
Schematic diagram of multiple compartments used in our computational model: Two compartments of “systemic plasma” and “tissues” for systemic effects of drug and the tumour compartment comprising of “tumour plasma”, “tumour extravascular extracellular space (EES)” and “tumour cells”. TSLs: Thermo-sensitive liposomes.

A schematic diagram of the computational model describing the interactions of multiple transport steps is shown in Figure 1. A multi-compartment modelling approach is employed to simulate the PK of TSLs and anticancer drugs at the systemic level as well as drug transport in the tumour compartment that is composed of tumour plasma, tumour extravascular extracellular space (EES) and tumour cells. The computational model has been improved upon our previous study [8] by accounting for the spatial distribution of free drug (bioavailable) in tumour interstitium, while drug-loaded TSLs are injected intravenously and triggered to release their encapsulated contents in response to HT exposure at the targeted tumour site. An idealised tumour cord geometry is employed to represent tumour vasculature for simplification and computational efficiency, which consists of a cylindrical annulus representing the capillary (with a radius *R_c_* = 10 μm) and its surrounding tumour interstitium (with a radius *R_t_* = 120 μm). Given the assumption of axis-symmetry and homogeneous distribution along tumour microvessels, one-dimensional drug transport in the radial direction is formulated.

HT exposure is the required external stimulus to trigger anticancer drug release from TSLs. Although the spatio–temporal distribution of temperature in tumour tissue can be predicted by coupling bio-heat transfer models (e.g., Pennes’ model) with drug transport models [6,9,10], the current study focuses on resolving drug distributions only by assuming uniform temperature in the tumour microenvironment (i.e., capillary and its surrounding interstitial space). This is justifiable when intense heating is applied via an external source and consequently a temperature increase occurs instantaneously. This simplification enables efficient computation, which is advantageous especially for model-based optimisation that involves numerous iterative steps.

The model equations are outlined below to explain major modifications from the previous model. Further details on the modelling approach and assumptions can be found in our previous work [8].

### 2.1. Systemic Drug Transport: Pharmacokinetics of TSLs and Anticancer Drug

As in previous studies [8,10], pharmacokinetics of TSLs and an anticancer drug and/or nanosized drug carrier can be described by a two-compartment model, with the assumption that only anticancer drug can move between the central (systemic plasma) and peripheral compartments (lumped tissue). Temporal variations in the systemic concentrations of TSL and anticancer drug and the tissue-level concentration of anticancer drug for bolus injection of TSL can be described by:

(1) $$V_{P}^{B}\frac{dc_{P,Lip}^{B}}{dt}=-k_{e,Lip}c_{P,Lip}^{B}V_{P}^{B}-kr_{37}c_{P,Lip}^{B}V_{P}^{B}+F_{PV}^{T}V_{P}^{T}c_{P,Lip}^{T}-F_{PV}^{T}V_{P}^{T}c_{P,Lip}^{B}$$

(2) $$V_{P}^{B}\frac{dc_{P}^{B}}{dt}=kr_{37}c_{P,Lip}^{B}V_{P}^{B}-k_{e}c_{P}^{B}V_{P}^{B}-k_{P}c_{P}^{B}V_{P}^{B}+k_{t}c_{t}^{B}V_{t}^{B}+F_{PV}^{T}V_{P}^{T}c_{P}^{T}-F_{PV}^{T}V_{P}^{T}c_{P}^{B}$$

(3) $$V_{t}^{B}\frac{dc_{t}^{B}}{dt}=k_{P}c_{P}^{B}V_{P}^{B}-k_{t}c_{t}^{B}V_{t}^{B}$$

where *V* is the volume of each compartment, *c* the concentration of TSL or drug, *kr*_37_ the drug release rate at the body temperature, *k_e_* the elimination rate from the central compartment, and *k_p_* and *k_t_* are the rate constants for drug transfer between the central plasma and tissue compartments. The subscripts *P*, *t*, *Lip* denote the plasma, tissue and liposome, respectively. The superscripts *B* and *T* represent the systemic level and the tumour compartment, respectively. $F_{PV}^{T}$ is the plasma flow per tumour plasma volume, defined as: $F_{PV}^{T}=w_{blood}\left(1-H_{ctt}\right)/v_{P}^{T}$ in which $w_{blood}$, $H_{ctt}$ and $v_{P}^{T}$ are the perfusion rate in (mL/s/mL), haematocrit and volume fraction of tumour plasma, respectively.

### 2.2. Drug Transport in the Tumour Compartment

#### 2.2.1. Transport in Tumour Plasma

The release of anticancer drug from TSLs in tumour plasma depends on temperature and concentration of TSLs, which can be described by the first-order kinetics with the release rate constants *kr*_37_ and *kr*_42_ at the body temperature and mild hyperthermia upon heating, respectively. The dynamic concentrations of TSLs and anticancer drug are described by:

(4) $$V_{P}^{T}\frac{dc_{P,Lip}^{T}}{dt}=F_{PV}^{T}V_{P}^{T}c_{P,Lip}^{B}-F_{PV}^{T}V_{P}^{T}c_{P,Lip}^{T}-R\cdot c_{P,Lip}^{T}V_{P}^{T}$$

(5) $$V_{P}^{T}\frac{dc_{P}^{T}}{dt}=F_{PV}^{T}V_{P}^{T}c_{P}^{B}-F_{PV}^{T}V_{P}^{T}c_{P}^{T}+R\cdot c_{P,Lip}^{T}V_{P}^{T}$$

(6) $$R=\;\{\begin{array}{c} kr_{42},\mathrm{for}\;T_{c}\leq t\leq \;T_{c}+T_{h}\\ kr_{37},\;otherwise \end{array}$$

where *R* is the release rate constant determined by temperature and *T_c_* and *T_h_* are the initial time and duration of HT, respectively.

#### 2.2.2. Transport in the Tumour Interstitium

Both diffusive and convective transport mechanisms are important in determining the accumulation of drug in tumour interstitium. Anticancer drugs are also taken up and pumped out by tumour cells, which can be modelled using the first-order kinetics and Michaelis–Menten kinetics for both passive diffusion and active transport. Therefore, the concentration of anticancer drug in tumour interstitium can be described by the convection-diffusion-reaction equation, whereas the transport of TSLs is assumed to be diffusion-dominated.

(7) $$\frac{\partial c_{e}}{\partial t}=D\nabla^{2}c_{e}-c_{t}\left(k_{1ci}c_{e}+\frac{V_{max}c_{e}}{c_{e}+K_{e}v_{e}^{T}}-\frac{V_{max}c_{i}}{c_{i}+K_{i}}\right)-u_{i}\cdot \nabla c_{e}$$

(8) $$\frac{\partial c_{i}}{\partial t}=k_{1ci}c_{e}+\frac{V_{max}c_{e}}{c_{e}+K_{e}v_{e}^{T}}-\frac{V_{max}c_{i}}{c_{i}+K_{i}}$$

(9) $$\frac{\partial c_{e,Lip}}{\partial t}=D_{Lip}\nabla^{2}c_{e,Lip}-R\cdot c_{e,Lip}$$

where *c_e_* is the extracellular concentration, *c_i_* the intracellular anticancer drug, *D* the diffusivity, $v_{e}^{T}$ the volume fraction of tumour EES, *k_1ci_* the rate constant for passive transport, *V_max_* the maximum rate for transmembrane transport, *K_e_* and *K_i_* the Michaelis constants, and *R* is the release rate constant defined in Equation (6). The blood flow velocity *u_i_* in the interstitial space is determined by the permeability of vessel walls (K) and pressure difference between the blood vessel and tumour interstitial space (*P_i_*) via Darcy’s law.

(10) $$u_{i}-\nabla \cdot \left(KP_{i}\right)=0$$

(11) $$\nabla u_{i}=0$$

Solving Equations (7), (10) and (11) requires boundary conditions for *c_e_* and *u_i_*. Starling’s law and Kedem–Katchalsky equations are used to prescribe the transmural blood flow velocity and drug flux at the capillary walls, i.e., at *r* = *R_c_*. It is assumed that osmotic pressure is negligible compared to the pressure gradient in determining the transmural velocity in solid tumour [4]. These boundary conditions are expressed as:

(12) $$u_{i}=J_{F}=L_{p}\left(P_{v}-P_{i}\right),\;\mathrm{at}\;r=R_{c}\;\mathrm{and}\;\mathrm{for}\;t>0$$

(13) $$-D\frac{\partial c_{e}}{\partial r}=P\left(c_{P}^{T}-c_{e}\right)+J_{F}\left(1-\sigma_{F}\right)\frac{c_{v}-c_{e}}{\ln\left(\frac{c_{v}}{c_{e}}\right)},\;\mathrm{at}\;r=R_{c}\;\mathrm{and}\;\mathrm{for}\;t>0$$

where *J_F_* is the transmural velocity, *P_v_* the vessel pressure, *c_v_* is the drug concentration in the vessel side, *L_p_* the hydraulic permeability and *σ_F_* the reflection coefficient. At the outer boundary of the tumour cord (*r* = *R_t_*), no flux is prescribed for *c_e_*as expressed in Equation (14).

(14) $$\frac{\partial c_{e}}{\partial r}=0,\;\mathrm{at}\;r=\;R_{t}\;\mathrm{and}\;\mathrm{for}\;t>0$$

### 2.3. Parameterisation and Initialisation

Values of parameters in the model equations are adopted from the literature or estimated from relevant in vitro experimental data (see Table 1). Parameters characterising TSLs, namely release rates at body temperature and during HT ($kr_{37}$ and $kr_{42}$), are strongly dependent on the TSL formulation. Release rates used in this study are estimated as approximation of linear kinetics by fitting to release curves of TOP-iTSLs [16], and the same values are used for DOX-TSL. Also, the elimination rate of both drugs from the systemic plasma is assumed the same, as their average half-life is similar, 2–3 h for TOP [17] and 1–3 h for DOX [18]. Other parameters for iTSLs are assumed to be identical to those for TSLs, found in the literature listed in Table 1. Finally, initial values for all variables are set to zero, except for TSLs concentration in systemic plasma for bolus injection, which is determined by $Dose/V_{P}^{B}.$

### 2.4. Numerical Methods

Spatial derivatives in the model equations are discretised via the finite difference methods. As a result, the partial differential equations become a set of ordinary differential equations to resolve temporal concentrations at each discretised segment. The system of ordinary differential equations for TSL and drug concentrations in each compartment is solved numerically by means of an in-built solver in MATLAB (ode15s). Mesh sensitivity tests are carried out and the final grid size is Δr=0.4 μm and time step Δt=0.1.

### 2.5. Optimisation

Using the developed mathematical model, an optimisation framework is established in order to identify the best drug release properties of TSL and heating schedule that can achieve reduced side effects and enhanced treatment outcome. Specifically, we aim to optimise the drug release rates (*kr*_37_ and *kr*_42_) and HT duration (*T_h_*) for maximised intracellular drug concentration (*c_i_*) and minimised systemic drug concentration (*c_B_^P^*) over time, which are indicative of therapeutic efficacy and the risk of side effects, respectively. For this purpose, a multiobjective optimisation problem is formulated, which can be solved by the weighted sum method and described as follows:–

(15) $$\begin{array}{l} \underset{kr_{37},kr_{42},T_{h}}{Minimise}\;\{\begin{array}{c} J_{1}=\frac{{\bar{c}}_{B}^{P}}{{\bar{c}}_{B,base}^{P}}\\ J_{2}=-\frac{{\bar{c}}_{i}}{{\bar{c}}_{i,base}} \end{array}=\underset{kr_{37},kr_{42},t_{h}}{Minimise\;}\left(w_{1}J_{1}+w_{2}J_{2}\right)\\ \mathrm{subject}\;\mathrm{to}\;w_{1}+w_{2}=1\\ \;\mathrm{Equations}\;(1)–\left(14\right)\\ \;kr_{37,lb}\leq kr_{37}\leq kr_{37,up}\\ \;kr_{42,lb}\leq kr_{42}\leq kr_{42,up}\\ \;T_{h,lb}\leq T_{h}\leq T_{h,up} \end{array}$$

where *J*_1_ and *J*_2_ are the objective functions to be minimised that represent time-averaged systemic plasma drug concentration and intracellular drug concentrations normalised by their respective baseline values (as defined in Table 1), and *w_i_* the weighting factor for the *i*-th objective function value. The model equations presented in Equations. (1) to (14) serve as equality constraints and each optimisation variable is bounded by its lower and upper limits defined as: 0.1*kr_37,ref_* ≤ *kr_37,ref_* ≤ 10*kr*_37_, 0.1*kr_42,ref_* ≤ *kr*_42_ ≤ 10*kr_42,ref_* and 0 ≤ *T_h_* ≤ 3600 s. The weighting factor *w*_1_ is varied between 0 and 1 and optimisation results for different weighting factors are compared. Also, multiple sets of initial guesses of the optimisation variables are chosen to ensure global optimality. The above optimisation problem is solved via an in-built function in MATLAB (fmincon), designed for constrained nonlinear multivariable problems.

## 3. Results and Discussion

Simulation results are presented for the baseline case first. This is followed by a comparison of two different anticancer drugs, and a parametric study of release rate constants and heating duration. Finally, optimisation of the key parameters is performed.

### 3.1. Simulation Results for the Baseline Scenario

The baseline case is designed to provide detailed information on spatio–temporal drug distributions in different compartments. We chose topotecan (TOP) loaded TSLs for the baseline case, as the release rate constants were derived from the experiments for TOP-iTSL [16]. In the baseline scenario, a bolus of 16 μg/mL TOP-iTSLs is intravenously injected into the systemic and tumour plasma, and heating is applied at 30 min for 3 min and at 90 min for 5 min after the treatment begins, following the work described in [16].

**Figure 2:**
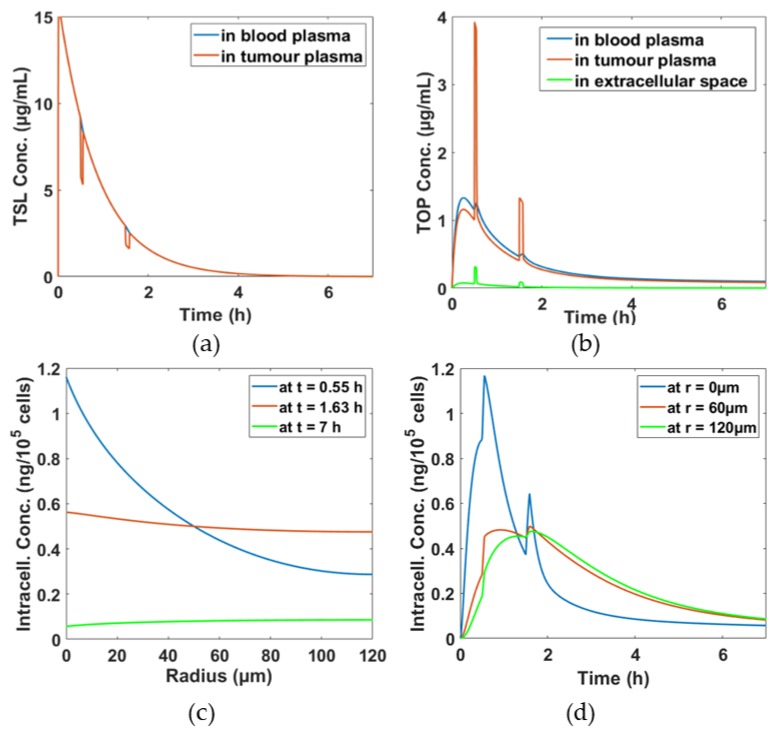
Spatio–temporal distributions of TSL and topotecan (TOP) concentrations in different compartments. (**a**) TSL concentrations in the systemic and tumour plasma compartments, (**b**) released TOP concentration in the systemic plasma, tumour plasma and extracellular space, (**c**) spatial profiles of TOP intracellular concentrations at different time points and (**d**) temporal profiles of TOP intracellular concentrations at different radial positions.

Figure 2a shows TSL concentrations in both systemic and tumour plasma gradually decline from its initial concentration over time mainly due to systemic clearance and transfer to other compartments. As TSLs move between the systemic plasma and tumour plasma compartments rapidly, an equilibrium of TSL concentration in both compartments is quickly established. However, there are sharp falls in TSL concentration in the tumour plasma compartment at 30 min and 90 min by approximately 4 μg/mL and 1 μg/mL, respectively, due to elevated temperature by heating and rapid breakdown of TSL at 42 °C. In Figure 2b, TOP concentrations in different compartments are displayed. During hyperthermia, a large amount of TOP is released, resulting in peaks of TOP level in the tumour plasm. This also affects the tumour extracellular and intracellular space, where concentration peaks also appear at the same time instants, as in Figure 2b,d, respectively. Furthermore, temporal distributions of TOP at different radial positions are presented in Figure 2d. It shows that TOP concentration near the tumour core is more sensitive to hyperthermia than at locations further away from the core, with the edge of the tumour being hardly influenced by hyperthermia, i.e., no concentration peaks at the time of heating. Figure 2c shows the spatial drug distributions at different time points, t = 0.55 h, t = 1.63 h and t = 7 h, the times immediately after the first hyperthermia, second hyperthermia, and the end of the treatment, respectively. As can be seen from both Figure 2c,d, TOP is not uniformly distributed within the tumour cord especially in the first hour of the treatment, with very high TOP concentration near the core close to the blood vessel (at *r* = 0). As time proceeds, TOP starts to distribute more uniformly.

This simulation represents an initial effort of using mathematical models to understand the micro-distribution of a bioavailabile drug following TSLs-mediated drug delivery. For validation purposes, the model predictions are compared with animal experiments performed by Centelles et al. [16] who tested TOP-loaded iTSLs on a murine cancer model. A good qualitative agreement can be found between the experimental results and the simulation results in capturing the sharp increase in TOP concentration after each hyperthermia (Figure 2b,d) in this work and Figure 5 in [16]); the murine cancer model also exhibited higher TOP-related fluorescence intensity immediately after hyperthermia was applied, indicating a burst release of TOP from iTSL. Quantitative comparisons are not made, because it was not possible to measure TOP concentrations in the experiment [16].

### 3.2. Comparison of DOX and TOP

The same model has been used to simulate DOX-TSL, assuming the same liposomal properties and heating duration. Hence, any difference in drug concentration would be mainly attributed to the different transport properties (i.e., diffusivity and permeability) that are estimated from their molecular sizes.

**Figure 3:**
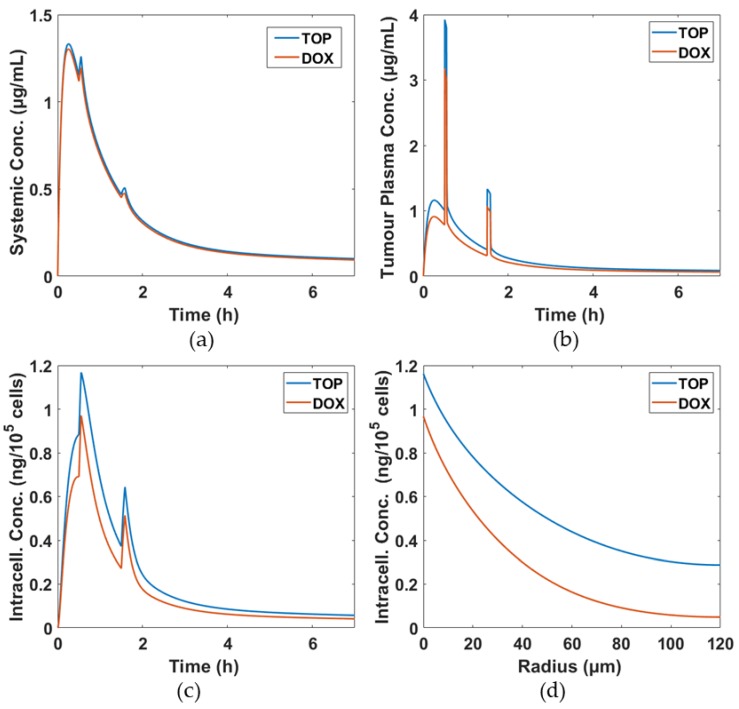
Simulation results for two different drugs, DOX and TOP. (**a**) Free drug concentrations in systemic plasma, (**b**) free drug concentrations in tumour plasma, (**c**) temporal intracellular drug concentrations in the intracellular space at *r* = 0 µm over the course of treatment and (**d**) spatial intracellular drug concentrations at *t* = 0.55 h.

Distributions of DOX and TOP concentrations in the systemic plasma, tumour plasma and intracellular space are shown in Figure 3. Due to the same release rate for TOP-TSL and DOX-TSL, there is hardly any difference in systemic drug concentration. In the tumour and intracellular compartments, DOX concentrations are lower than TOP concentrations, meaning that more TOP can reach tumour cells. This is because TOP has a smaller molecular size than DOX, which results in higher diffusivity and permeability. These results reiterate that small molecule drugs would transport more easily through the tumour without increasing their exposure at the systemic level [21,22,23].

### 3.3. Effects of Drug Release Rates

**Figure 4:**
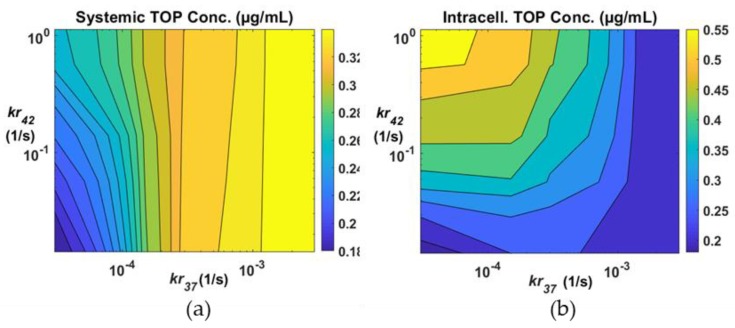
Drug concentration averaged over the course of the treatment with different combinations of release rate constants *kr*_37_ and *kr*_42_ (**a**) in the systemic plasma and (**b**) in intracellular space.

The rate of drug release from TSL is temperature dependent and varies with the liposome formulation. In our current model, two release constants are required: One at physiological temperature (37 °C) and another at mild hyperthermia (42 °C). We chose TOP as the anticancer agent for simulations presented hereafter. In Figure 4, drug concentrations in the systemic plasma and tumour intracellular compartments are shown with different combinations of release rate constants. The systemic drug concentration displayed in Figure 4a has a direct impact on the risk of side effects of the treatment, whereas the intracellular drug concentration shown in Figure 4b is considered representative of therapeutic efficacy. The contour maps show that a low value of *kr*_37_ would be needed in order to keep the systemic concentration low. On the other hand, a high *kr*_42_ would be desirable for enhanced cancer cell killing. Results in Figure 4b show that intracellular drug concentration is highly sensitive to *kr*_42_, but its effect on systemic concentration is rather trivial, especially at moderate and high *kr*_37_.

The extent of drug leakage at body temperature is controlled by *kr*_37_ [24]. Ideally, this should be kept as low as possible in order to achieve controlled drug release trigged by hyperthermia. As *kr*_37_ increases, more encapsulated drugs would be released before hyperthermia is applied, rendering the process less temperature sensitive. The rate of drug release at hyperthermia is controlled by *kr*_42_. A very high *kr*_42_ value corresponds to instantaneous drug release, which triggers a sharp rise in free drug concentration in the tumour plasma compartment upon hyperthermia, as shown in Figure 2b and Figure 3b. This would lead to a significant difference in drug concentration between the tumour plasma and extracellular space, providing substantial driving force for diffusive drug transport into the intracellular space.

### 3.4. Effects of Hyperthermia (HT) Duration

Simulations are undertaken for different heating schedules while keeping the release rate constants unchanged. Three additional heating schedules are simulated: A two-step heating of 12 min and 20 min, a two-step heating of 24 min and 40 min and a single stage heating for 64 min. The two-step heating schedules consist of a short heating duration followed by a longer heating duration, following the heating protocol adopted in the experimental work [16]. The time points when hyperthermia is applied are also kept the same as in the baseline case, i.e., at 30 and 90 min from the start of the treatment.

**Figure 5:**
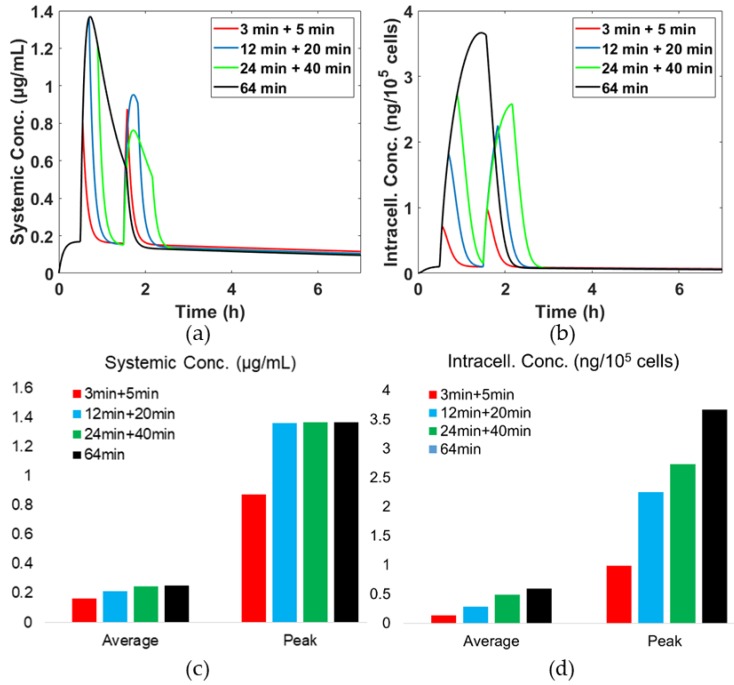
Temporal concentrations of drug with different hyperthermia schedules. (**a**) Free drug concentration in systemic plasma, (**b**) drug concentration in intracellular space at *r* = 0 µm, (**c**) average and peak drug concentration in systemic plasma and (**d**) average and peak topotecan concentration in intracellular space at *r* = 0 µm.

Figure 5 displays drug concentrations in the systemic plasma and intracellular space. It is interesting all heat schedules achieve the same peak systemic drug concentration of 1.4 μg/mL at the first hyperthermia, as depicted in Figure 5a. However, upon application of the second hyperthermia, different peak values at achieved, at between 0.75 and 1 μg/mL. The longer the first heating, the lower the drug concentration reached during the second heating. This is due to the remaining amount of encapsulated drug being different after the first hyperthermia. Figure 5b displays the temporal variation of intracellular drug concentration. The longer the heating duration, the higher the intracellular drug concentration, e.g., when the heating time is increased by 8 times relative to the baseline case, there is almost a 4-fold increase in the intracellular drug concentration. This means that an extended heating duration might be beneficial in enhancing the treatment efficacy due to high intracellular drug concentration.

Figure 5c,d give the average and peak concentrations in the systemic plasma and intracellular space. It shows that the single heating protocol achieves the highest peak and average concentrations in both the systemic plasma and intracellular compartments. When the single heating protocol is compared with the two-step heating protocol of the same total heating duration, i.e., 24 min + 40 min, there is no difference in the systemic drug concentration, but the level of drug in the intracellular space is higher with the single heating than the two-step heating protocol. This indicates that the risk of cytotoxicity might not be affected by the choice of heating protocols as long as the total heating duration is the same, whereas the therapeutic efficacy could be enhanced by continuous heating.

### 3.5. Optimisation Results

An initial optimisation exercise is performed for TOP-loaded TSL delivery with a single heating protocol. Three optimisation variables, *kr*_37_, *kr*_42_ and *T_h_*, are simultaneously manipulated during the computer-based optimisation until the objective function value, the weighted sum of systemic plasma and intracellular concentrations as described in Equation (15), reaches its minimum. The weighting factors are also varied for global optimisation, and optimisation results are compared in Table 2. The higher the weighting factor *w*_1_, the more we value the minimisation of systemic drug concentration, and vice versa. As the weighting factor, *w*_1_, changes from 0 to 1, the optimisation problem turns from the maximisation of intracellular concentration to the minimisation of systemic plasma concentration. Table 2 indicates that regardless of the release constants and weighting factor, the optimal heating duration is found to be at its upper limit, 3600 s. Also, a similar behaviour for *k_37_* can be seen; the optimal *k_37_* value is at its lower bound regardless of the weight factor and *k_42_*, which means that a low release rate at 37 °C is always beneficial. On the other hand, the optimal value for *k_42_* depends strongly on the weight factor. As *w*_2_ increases (i.e., only maximising the intracellular drug concentration), *kr*_42_ approaches its upper bound. When the weight factors are equal (i.e., *w*_1_ = *w*_2_ = 0.5), a moderate *kr*_42_ would be optimal. However, the impact of *kr*_42_ on systemic drug concentration is weak, and the rate of increase in intracellular concentration slows down significantly after *kr*_42_ reaches 0.7 s^−1^, suggesting that further increase in *kr_42_*would not enhance intracellular concentration any further. It is worth noting that our optimisation results have limitations especially arising from the current formulation of objective function; the average systemic and intracellular drug concentrations are used to indicate the extent of cytotoxicity and cancer cell killing efficacy, respectively. The concentration terms in the objective function can be replaced by more direct and/or accurate predictors of cytotoxicity and cancer cell killing efficacy. In addition, the optimisation is based on the assumption of uniform temperature in tumour interstitium.

## 4. Conclusions and Future Perspectives

TSL-mediated drug delivery in combination with hyperthermia exposure has been considered as a promising alternative to conventional chemotherapeutics. The transport of TSL and its encapsulated drug from systemic circulation to tumour cells involves multiple steps and non-linear dynamic interactions. In the current study, mathematical models have been developed to describe the multiple transport processes involved in TSL-DOX/TOP systems. Computational simulations have been carried out to reveal micro-scale distributions of anticancer drugs in a tumour cord model following a bolus injection of TSL and upon application of hyperthermia. The impacts of TSL release rates, heating duration and type of drugs (DOX vs. TOP) are also investigated. Finally, we have undertaken model-based optimisation aimed at maximising intracellular drug concentration in tumour, while minimising the systemic plasma concentration of the drug.

Our simulation and optimisation results have provided more insights into the role of several key factors in determining the efficacy of TSL-mediated drug delivery; these include parameters related to the TSL formulation, properties of encapsulated drugs and HT exposure duration. The modelling and optimisation framework described here is expected to serve as a useful tool in the design of TSLs and treatment schedules for various TSL-mediated therapies. It can be particularly useful in identifying the key parameters that can be controlled to improve the performance of a drug delivery system, and in complementing experimental research through parametric sensitivity analysis and optimisation.

The model can be further improved by incorporating bioheat transfer equations, so as to accommodate the effect of heterogeneous temperature distribution. The current model for microvascular transport can be extended relatively easily to incorporate fluid flow and intravascular drug transport, as demonstrated by Liu et al. [25]. In addition, specific cell killing models for different type of cancer cells and anticancer drugs could be employed to make the prediction more tumour specific.

## Figures and Tables

**Table 1:** Parameters and baseline values used in the computational model.

Symbol	Description	Value	Reference
$k_{e,Lip}$	Rate constant of TSLs clearance	9.417 × 10^−6^ (1/s)	[9]
$k_{e}$	Rate constant of drug clearance	1.1 × 10^−3^ (1/s)	[6]
$k_{p}$	Transfer constant from systemic plasma to tissue	1.6 × 10^−3^ (1/s)	[6]
$k_{t}$	Transfer constant from tissue to systemic plasma	4.68 × 10^−5^ (1/s)	[6]
$kr_{37}$	Release rate constant from iTSLs at body temperature	3 × 10^−4^ (1/s)	[16]
$kr_{42}$	Release rate constant from iTSLs during HT (at 42 °C)	0.114 (1/s)	[16]
$K_{e}$	Michaelis constant for transmembrane transport	2.19 × 10^−4^ (µg/mm^3^)	[19]
$K_{i}$	Michaelis constant for transmembrane transport	1.37 (ng/10^5^ cells)	[19]
$V_{\mathrm{ma}x}$	Maximum rate for transmembrane transport	0.28 (ng/(10^5^ cells·min))	[19]
$k_{1ci}$	Rate for passive intracellular uptake	6.33 × 10^−4^ (1/s)	Fit to [6]
$V_{p}^{B}$	Volume of systemic plasma	3.04 (L)	[9]
$V_{t}^{B}$	Volume of body tissue	64.47 (L)	[9]
$V^{T}$	Volume of tumour tissue	8.82 × 10^−2^ (L)	Estimated based on a spherical tumour with a radius of 2.7 cm
$v_{p}^{T}$	Volume fraction of tumour plasma	0.07452	[9]
$v_{e}^{T}$	Volume fraction of tumour EES	0.454	[9]
$v_{i}^{T}$	Volume fraction of intracellular space	0.454	[9]
$w_{blood}$	Blood perfusion rate	0.018 (1/s)	[9]
$H_{ctt}$	Haematocrit for tumour microvasculature	0.19	[9]
$PS_{t}$	Permeability surface area product for drugs	2.53 × 10^−3^ (1/s) (TOP) 7 × 10^−3^ (1/s) (DOX)	Estimated based on molecular size [9]
$PS_{L}$	Permeability surface area product for TSL	4.76 × 10^−6^ (1/s) (TOP) 2.38 × 10^−5^ (1/s) (DOX)	Estimated using the $PS_{t}$, P and $P_{lip}$ of DOX and TOP respectively
P	Diffusive permeability for drugs	3.61 × 10^−7^ (m/s) (TOP) 1 × 10^−6^ (m/s) (DOX)	Estimated based on molecular size [19]
$P_{Lip}$	Diffusive permeability for TSLs and iTSLs	3.4 × 10^−9^ (m/s)	[19]
$D_{}$	Diffusion coefficient	4.123 × 10^−10^ (m^2^/s) (TOP) 1.578 × 10^−10^ (m^2^/s) (DOX)	Estimated based on molecular size [12]
$D_{Lip}$	Diffusion coefficient for TSLs and iTSLs	9 × 10^−12^ (m^2^/s)	[20]
$R_{c}$	Tumour capillary radius	10 (μm)	[12]
$R_{t}$	Tumour cord radius	120 (μm)	[12]
$V_{c}$	Volume of single tumour cell	1 × 10^−6^ (mm^3^/cell)	[19]
Dose	Total dose	49 (mg)	Calculated at a dose of 0.7 mg/kg in a 70 kg human

**Table 2:** Optimisation results with different weighting factors in the objective function.

Weighting Factor, *w*_1_, for the Systemic Plasma Conc.	Weighting Factor, *w*_2_, for the Intracellular Conc.	*kr*_37_ (10^−4^ s^−1^)	*kr*_42_ (s^−1^)	Hyperthermia Duration (s)	Systemic Plasma Concentration (μg/mL)	Intracellular Concentration (ng/10^5^ cells)
0	1	0.3	1.14	3600	0.26	0.56
0.25	0.75	0.3	1.1	3600	0.25	0.56
0.5	0.5	0.3	0.7	3600	0.24	0.55
0.75	0.25	0.3	0.13	3600	0.21	0.48
1	0	0.3	0.0114	3600	0.18	0.19
